# A preliminary study of the effect of curcumin on the expression of p53 protein in a human multiple myeloma cell line

**DOI:** 10.3892/ol.2015.2946

**Published:** 2015-02-09

**Authors:** WEI LI, YAOMEI WANG, YONGPING SONG, LINPING XU, JUNMEI ZHAO, BAIJUN FANG

**Affiliations:** 1 Department of Immunotherapy, The Affiliated Cancer Hospital of Zhengzhou University, Zhengzhou University, Zhengzhou, Henan, P.R. China; 2 School of Life Sciences, Zhengzhou University, Zhengzhou, Henan, P.R. China; 3 Laboratory of Membrane Biology, New York Blood Center, New York, NY, USA; 4 Department of Hematology, The Affiliated Cancer Hospital of Zhengzhou University, Zhengzhou, Henan, P.R. China

**Keywords:** curcumin, multiple myeloma, p53 protein

## Abstract

Curcumin is an inexpensive, natural plant ingredient with protease inhibitor effects. The present study aimed to analyze the inhibitory effects of curcumin on the multiple myeloma (MM) RPMI 8226 cell line, and examine the underlying mechanism that promotes the apoptosis of RPMI 8226 cells. A growth curve was constructed in order to observe the relative growth velocity, and MTT was used to analyze the effect of different concentrations of curcumin on inhibiting the proliferation of the RPMI 8226 cells. The mRNA expression of the *p53*, *Bax* and *MDM2* genes was detected using quantitative polymerase chain reaction. The expression of p53 protein in the MM RPMI 8226 cells following treatment with curcumin was detected by western blotting and ELISA. Curcumin inhibited the proliferation of the MM RPMI 8226 cells in a dose- and time-dependent manner. In the MM RPMI 8226 cells treated with curcumin, the expression of the *p53* and *Bax* genes was upregulated, while the expression of the *MDM2* gene was downregulated. p53 protein expression was higher in the curcumin experimental group compared with the control group. Subsequent to treatment with curcumin, the growth of the MM RPMI 8226 cell line was inhibited in a concentration- and time-dependent manner. In the MM RPMI 8226 cells treated with curcumin, p53 protein levels were upregulated, which suggested that curcumin may promote the apoptosis of MM cells by upregulating p53 protein expression.

## Introduction

Multiple myeloma (MM) is a type of plasma cell-derived malignancy, which leads to the formation of multiple bone lesions and to disruption in the production of normal blood cells (1). MM accounts for ~10% of all hematological malignancies and is the second most common type of hematological malignancy after non-Hodgkin’s lymphoma (2). Although a number of therapeutic strategies exist, such as the use of steroids, chemotherapy, radiotherapy and stem cell transplants, MM remains an incurable disease (3). Patients with MM exhibit elevated levels of circulating proteasome. Therefore, it has been suggested that circulating proteasome levels may serve as an independent prognostic factor for the survival rates of patients with MM, and that proteasome therapy may be an effective treatment approach (4). The first proteasome inhibitor to be approved for clinical use by the US Food and Drug Administration (FDA) was bortezomib. Bortezomib, a reversible proteasome inhibitor, is approved by the FDA for treating refractory, advanced or rapidly relapsed cases of MM (5). Curcumin is a natural product with proteasome inhibitory effects that has been studied in a number of cancers, alone and in combination with other traditional chemotherapy and radiotherapy agents (6–8).

Curcumin is a primary active ingredient derived from the spice, turmeric. Curcumin suppresses tumor growth and inhibits cellular proliferation, invasion, angiogenesis, metastasis and osteoclastogenesis, which are processes that involve multiple cellular targets, such as nuclear factor (*NF*)-*κB* and *cyclooxygenase-2* (9,10). Curcumin is therefore considered to be a multi-targeted drug that suppresses *NF-κB* activation and reduces MM cell growth and apoptosis. Curcumin inhibits the proliferation, invasion, metastasis and angiogenesis of a number of cancers through interaction with a variety of cell signaling proteins (11), the majority of which are proteasome target proteins, such as the tumor suppressor protein, p53, and the pro-apoptotic protein, B-cell lymphoma 2 (Bcl-2) associated X protein (Bax) (12,13).

The tumor suppressor protein, p53 (13), and the pro-apoptotic protein, Bax (12), are proteasome target proteins, which are involved in the processes of cancer survival and carcinogenesis. Despite mutations in the *p53* gene occurring in 50% of all cancers, ~90% of MM cells retain a functional wild-type *p53* (14–17). The low incidence of mutations and deletions in the *p53* gene make MM an ideal candidate for p53-targeted therapies. Even in tumors that retain wild-type *p53*, *p53* function is ultimately inhibited by the action of mouse double minute 2 homolog (MDM2) (17–19). The life cycle of the p53 protein is short; during periods of cellular stress, the p53 protein is regulated by a negative feedback mechanism. Under non-stressful conditions, *p53* is regulated by the negative regulator, *MDM2*. Therefore, *p53* and *MDM2* form a feedback loop with each other and are maintained at low levels (20).

In the present study, the proliferation rate of the MM RPMI 8226 cell line was analyzed following treatment with curcumin. In addition, changes in the expression of the *p53*, *Bax* and *MDM2* gene fragments, and in the p53 protein were examined. Furthermore, the underlying mechanism by which curcumin promotes RPMI 8226 cell apoptosis was discussed for the application of curcumin in patients with MM.

## Materials and methods

### Cell growth curve

In total, RPMI 8226 cells (10^5^/ml; School of Life Sciences, Zhengzhou University, Zhengzhou, China) were seeded into six-well plates and cultured in triplicate in RPMI-1640 medium (Invitrogen Life Technologies, Carlsbad, CA, USA) containing 10% fetal bovine serum (Invitrogen Life Technologies), with or without curcumin. The final concentrations of curcumin were 0, 1, 2.5, 5, 7.5, 10, 15, 20 and 40 μmol/l. The final volume of medium in each well following the addition of curcumin was 1 ml. The number of cells in each well was counted every 24 h, and the cells were cultured as usual with the same medium until 96 h.

### MTT

First, 10^5^/ml RPMI-8226 cells were seeded into 96-well plates and cultured in six repeated wells. The experimental groups contained the cells and RPMI-1640 medium with 10% fetal bovine serum and curcumin. The positive control groups contained the cells and RPMI-1640 medium with 10% fetal bovine serum. The negative control groups contained RPMI-1640 medium alone with 10% fetal bovine serum. The final concentrations of curcumin were 1, 2.5, 5, 7.5, 10, 15, 20 and 40 μmol/l. The final volume of medium in each well following the addition of curcumin was 200 μl. Next, the surrounding wells were covered with 200 μl phosphate-buffered saline (PBS). The plates were then incubated at 37°C with 5% CO_2_ for 24, 48 and 72 h. Following this, 20 μl MTT solution (5 mg/ml in PBS, Sigma-Aldrich, Santa Clara, CA, USA) was added to each well of the experimental, and positive and negative control groups. Subsequent to a 4-h incubation at 37°C and subsequent centrifugation at 1,000 × g for 5 min at 37°C, 200 μl solution from every well was extracted, and 150 μl dimethylsulfoxide was added to the wells. After 15 min, the optical density (OD) at 490 nm was measured using an iMark microplate absorbance reader (Bio-Rad 550; Bio-Rad Laboratories, Inc., Hercules, CA, USA). The cell proliferation inhibition ratio was calculated using the following formula: Cell proliferation inhibition ratio = 1- (A490 of the experimental groups / A490 of the control groups) × 100. The A490 of the experimental groups = OD of the experimental groups - OD of the negative control groups. The A490 of the positive control groups = OD of the positive control groups - OD of the negative control groups.

The average half maximal inhibitory concentration of curcumin from six experiments was obtained by plotting the percentage of inhibition against the concentration of curcumin.

### Polymerase chain reaction (PCR)

In total, 10^6^/ml RPMI-8226 cells in the logarithmic phase were seeded into 25-cm^2^ culture bottles for the positive control and experimental groups, including the 10- and 15-μmol/l curcumin groups. The final volume of solution in each culture bottle was 5 ml. The positive control groups contained the cells and RPMI-1640 medium with 10% fetal bovine serum. The experimental groups contained the cells, curcumin and RPMI-1640 medium with 10% fetal bovine serum. The total RNA was isolated after 48 h from the cells in the culture bottle using TRIzol reagent (Invitrogen Life Technologies). Next, the RNA was reverse transcribed into cDNA and quantitative PCR (qPCR) was performed using the two-step method. Briefly, 25 μl reaction volume consisting of 12.5 μl of 2X PCR Buffer for KOD FX [ a PCR amplification enzyme (Qiagen, Venlo, Netherlands)], 5 μl 2 mM dNTPs, 2 μl of each primer, 0.1 μl KOD, 2.4 μl water and 1 μl DNA. The standard conditions for PCR were as follows: 95°C for 2 min, followed by 40 cycles at 95°C for 30 sec, 62°C for 1 min, and a final extension at 72°C for 5 min. All reactions were performed in a PerkinElmer 2400 thermocycler (Perkin Elmer Applied Biosystems, Foster City, CA, USA). The 2^−ΔΔCt^ method was used to indicate the association between the expression of the target gene in the experimental group and the expression of the target gene in the positive control group. The sequences of the primers are shown in Table I.

### Western blot analysis

For the western blot analysis, the cells were harvested and lysed, and the proteins were separated using a 12.5% SDS-PAGE gel. The proteins were then transferred to a Hybond-C membrane (Invitrogen Life Technologies). Next, the membrane was blocked with Blotto A (5% blocking grade dry milk in Tris-buffered saline and Tween 20; Invitrogen Life Technologies) and probed using a monoclonal mouse anti-human p53 primary antibody (dilution, 1:300; Santa Cruz Biotechnology, Inc., Dallas, TX, USA) in Blotto A. The cells were incubated with the primary antibody at 4°C overnight, and then with monoclonal goat anti-mouse IgG-horseradish peroxidase-tagged secondary antibody (dilution, 1:1,000; Santa Cruz Biotechnology, Inc.) at room temperature for 1 h. Detection of chemiluminescence was conducted using an enhanced chemiluminescence western detection reagent (GE Healthcare Bio-Sciences, Pittsburgh, PA, USA) and developed on a BioMax XAR film (Kodak, Rochester, NY, USA).

### ELISA

In total, 0.5×10^6^/ml RPMI-8226 cells in the logarithmic phase were seeded into 6-well plates and cultured in four repeated wells. Control groups, and experimental groups, including 10 and 15 μmol/l curcumin groups, were used. The final volume of solution in each well was 1 ml. The control groups contained the cells and RPMI-1640 medium with 10% fetal bovine serum. The experimental groups contained the cells, curcumin and RPMI-1640 medium with 10% fetal bovine serum. The total protein was isolated after 48 h from the cells in each well of the plates using 100 μl lysis buffer with 1 mM EDTA, according to the manufacturer’s instructions. The ELISAs were conducted using the p53 kit (RAB0500, Sigma-Aldrich). The standard and sample groups were set, and the indicated reagents were added for the indicated time period according to the manufacturer’s instructions. The OD of each well was measured at 490 nm using a microplate reader. A standard curve was constructed according to the OD values of the standard groups and a formula was generated based upon this standard curve. The p53 protein concentration of each sample was calculated according to the formula: p53 concentration = 2318.3OD_value_ − 241.19.

### Statistical analysis

SPSS software version 17.0 (SPSS Inc., Chicago, IL, USA) was used to perform the statistical analysis. Data from the control and experiment groups were analyzed by an independent sample t-test. P<0.05 was considered to indicate a statistically significant difference.

## Results

### Growth curve

**Figure 1:**
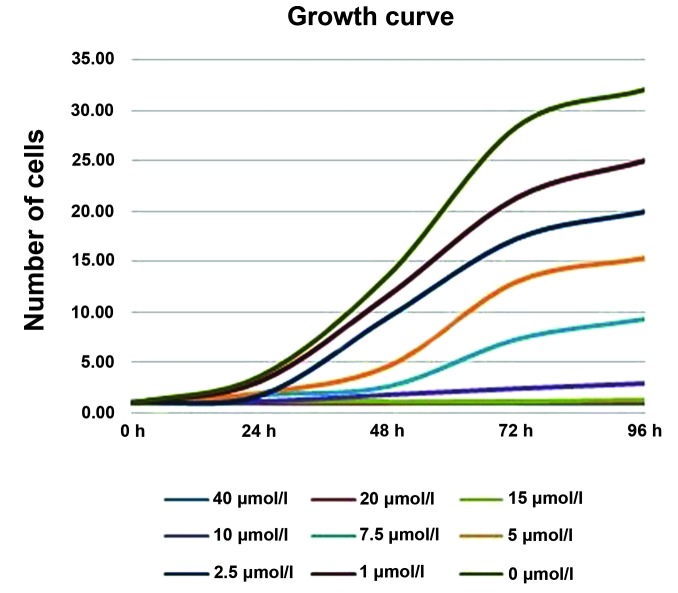
Growth curve representing the growth of cells treated with different concentrations of curcumin. The initial number of cells in each well was the same (10^5^/well). After 96 h, the number of cells in the wells treated with 0, 1, 2.5, 5, 7.5 and 10 μmol/l curcumin were >3×10^6^, ~2.5×10^6^, ~2×10^6^, ~1.5×10^6^, <1×10^6^ and <5×10^5^, respectively. The cells treated with 15, 20 and 40 μmol/l curcumin, however, barely grew.

The results revealed that the cells were in the logarithmic phase between 24 and 72 h. During this time period, the number of cells in the logarithmic phase decreased with increasing concentrations of curcumin. During the same period, the growth of cells treated with higher curcumin concentrations was slower (Fig. 1).

### MTT

**Figure 2:**
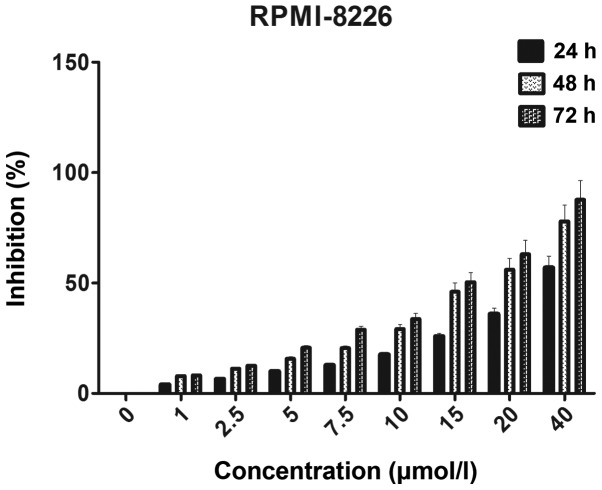
Cells were treated with various concentrations of curcumin for 24, 48 and 72 h prior to the determination of cytotoxicity by an MTT cell proliferation assay. Each value is expressed as the mean ± standard deviation of six measurements.

In order to investigate the effect of curcumin on the proliferation inhibition of the MM RPMI 8226 cell line, the OD was measured at 490 nm following 24, 48 and 72 h of treatment with different concentrations of curcumin. The proliferation inhibition ratio following 24, 48 and 72 h of treatment with 10 μmol/l curcumin was 17.6, 29.2 and 33.8%, respectively. The difference in the OD value was statistically significant between the experimental and control groups (P<0.05). The proliferation inhibition ratio following 24, 48 and 72 h of treatment with 15 μmol/l curcumin was 25.8, 46.1 and 50.4%, respectively. The difference in the OD value was statistically significant between the experimental and control groups (P<0.05). The results revealed that a higher concentration of curcumin was more potent than a lower concentration of curcumin at the same time-point in the growth suppression of the RPMI 8226 cells, and that a longer duration of treatment was more potent than a shorter duration of treatment with the same concentration of curcumin in the growth suppression of the RPMI 8226 cells (Fig. 2).

### qPCR

**Figure 3:**
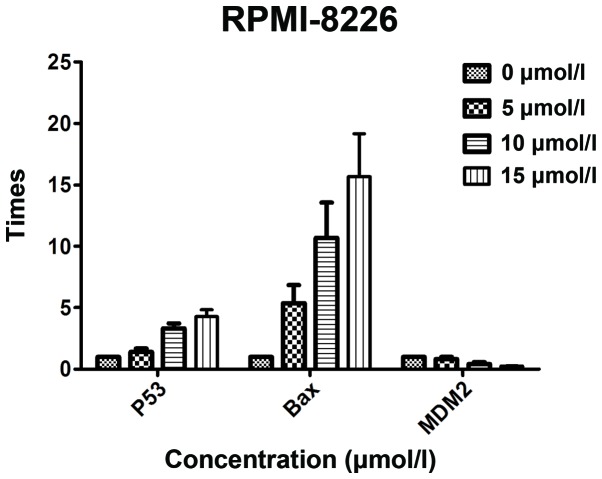
*p53*, *Bax* and *MDM2* gene fragment expression in the RPMI 8226 cells following treatment with different concentrations of curcumin for 48 h, as detected by polymerase chain reaction.

The qPCR results revealed that curcumin inhibited the growth of MM cells in a dose-dependent manner. Following a 48-h treatment with 10 and 15 μmol/l curcumin, the proliferation inhibition ratio was 29.2 and 46.1%, respectively. It has been reported that curcumin regulates the expression of the apoptosis-related proteins, Bax, Bcl-2 and p53, and that it regulates the apoptosis of tumor cells via the *p53* pathway (21). The low incidence of mutations and deletions in the *p53* gene make MM an ideal candidate for p53-targeted therapies (14–17). In order to investigate whether curcumin inhibits the growth of MM cells through the *p53* pathway, qPCR was used to analyze the expression of *p53*, *Bax* and *MDM2* gene fragments following treatment with different concentrations of curcumin. Subsequent to a 48-h treatment with 10 μmol/l curcumin, the expression of the *p53*, *Bax* and *MDM2* genes was 1.3905, 10.3581 and 0.4046 times higher than the expression of *p53*, *Bax* and *MDM2* in the control group, respectively. Furthermore, following a 48-h treatment with 15 μmol/l, the differences were 2.0871, 12.6826 and 0.2505 times higher. The differences were statistically significant (P<0.05). The results are shown in Fig. 3.

### Western blot analysis

**Figure 4:**
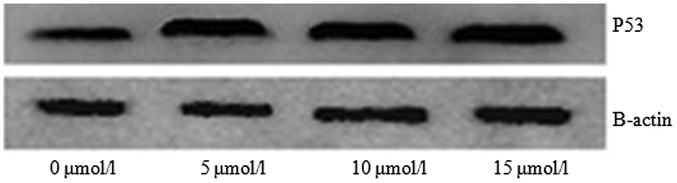
p53 protein expression detected by western blotting following treatment with different concentrations of curcumin for 48 h.

The *p53* signaling pathway is an important pathway involved in tumor cell apoptosis. When curcumin was used to treat the RPMI 8226 cells, the expression of the *p53* gene was increased compared with the control group. In order to detect the expression of the p53 protein following treatment with curcumin, a western blot assay was performed. It was revealed that the expression of the p53 protein in the cells treated with 5, 10 and 15 μmol/l curcumin for 48 h was increased compared with the control group (Fig. 4).

### ELISA

**Figure 5:**
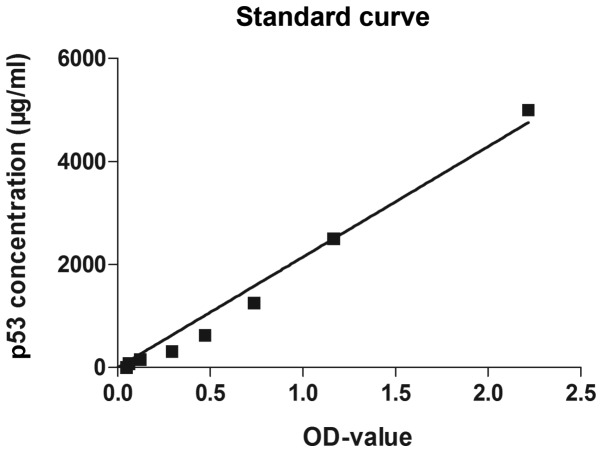
Measured optical density (OD) values of the standard samples were constructed as horizontal ordinates, and the concentrations of each diluted standard sample were contructed as vertical coordinates. The standard curve was drawn according to the OD values of the standard groups and the formula was then generated based on the standard curve.

**Figure 6:**
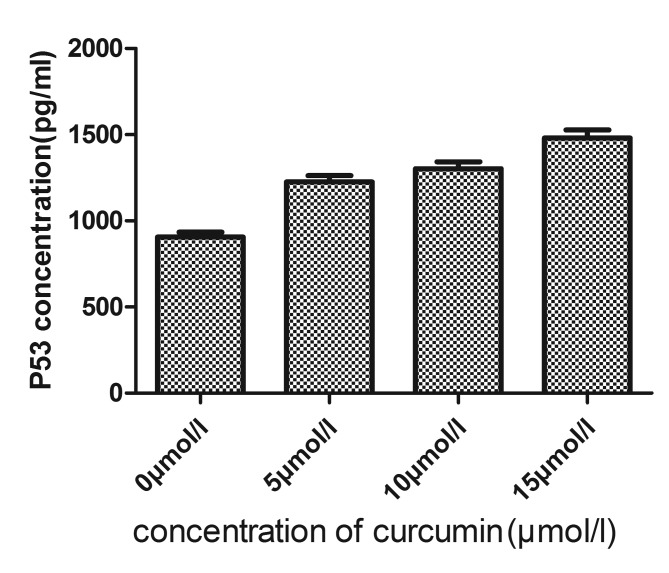
p53 protein concentration of each sample was calculated according to the formula: p53 concentration = 2318.3ODvalue − 241.19.

As the expression of the *p53* gene fragment was increased in the RPMI 8226 cells treated with curcumin, the present study next sought to determine the effect of curcumin on p53 protein expression in the MM RPMI 8226 cell line. The RPMI 8226 cells were treated with 5, 10 and 15 μmol/l curcumin for 48 h. The total proteins were then isolated and the expression level of the p53 protein was determined. The results revealed that the expression of the p53 protein was upregulated following treatment with curcumin in a dose-dependent manner (Table II; Figs. 5 and 6). These results indicated that curcumin promotes p53 protein expression, and may induce apoptosis through the *p53* pathway.

## Discussion

MM is a B-cell malignancy whereby plasma cells grow abnormally in the bone marrow and secret monoclonal immunoglobulin or an M protein fragment, which ultimately leads to relative organ or tissue injury. Although MM is sensitive to a variety of cytotoxic drugs in the initial and relapsed treatment periods, the relief is only temporary. Therefore, MM remains an incurable disease. A number of drugs, including bortezomib, a proteasome inhibitor, and thalidomide, an inhibitor of tumor necrosis factor production (22,23), have been tested during the search for an effective treatment for MM. Bortezomib, the first reversible proteasome inhibitor approved by the US FDA for treating refractory, advanced or rapidly relapsed MM (5), has been researched extensively. However, bortezomib is expensive, and the majority of patients with MM cannot afford to be treated with it. Curcumin is an inexpensive, natural plant ingredient with protease inhibitor effects, which has been studied, alone or in combination with traditional chemotherapy and radiotherapy agents against a number of cancers (6–8) Therefore, the present study analyzed the inhibitory effects of curcumin on the MM RPMI 8226 cell line, and examined the underlying mechanism that promotes the apoptosis of RPMI 8226 cells.

The results of the present study revealed that following treatment with curcumin, the growth of the MM RPMI 8226 cell line was inhibited in a concentration- and time-dependent manner, which was in agreement with the results of a study by Bharti *et al* (24). In the RPMI 8226 cells treated with curcumin, the expression of p53 protein was upregulated, which suggested that curcumin may promote the apoptosis of MM cells by upregulating p53 protein expression. A number of previous *in vitro* and *in vivo* studies have indicated that curcumin exhibits a variety of pharmacological effects, including antitumor, anti-inflammatory and antioxidant activities, and that the side-effects of treatment are minor (25). Other studies have revealed that curcumin can inhibit the proliferation of MM cells by downregulating the expression of interleukin-6 and *NF-κB*. Curcumin also prevents osteoclast-inducing osteogenesis and improves the resistance of MM cells to conventional chemotherapy drugs (26). Bharti *et al* (24) confirmed that curcumin can promote and induce the apoptosis of MM cells (24), and suppress osteoclastogenesis by inhibiting the receptor activator of *NF-κB* ligand signal (27). Therefore, curcumin exerts its antitumor effect through the mechanisms of inhibiting the proliferation of tumor cells, regulating the expression of oncogenes and anticancer genes, and inducing cell cycle arrest and apoptosis.

Curcumin also inhibits the proliferation, invasion, metastasis and angiogenesis of a number of cancers by interacting with a variety of cell signaling proteins (11), the majority of which are proteasome target proteins, including the tumor suppressor protein, p53, and the pro-apoptotic protein, Bax (12,13). The pro-apoptotic protein, Bax (12), and the tumor suppressor protein, p53 (13), are two types of proteasome target proteins involved in the processes of cancer survival and carcinogenesis. It has been reported that curcumin effects the expression of the apoptosis-related proteins, Bax, Bcl-2 and p53, and that it has the ability to regulate the apoptosis of tumor cells via the *p53* pathway (21). Furthermore, previous studies have demonstrated that curcumin can induce apoptosis through *p53*-dependent and -independent pathways during the treatment of endometriosis (28), and that it can upregulate the expression of p53 protein and *Bax* mRNA in thioacetamide-induced liver fibrosis (29). Therefore, it was hypothesized that curcumin may inhibit the proliferation and induce the apoptosis of MM cells through a *p53*-mediated pathway, which may be a novel therapeutic target for clinical use.

Despite mutations in the *p53* gene occurring in 50% of all cancers, ~90% of MM cells retain functional wild-type p53 (14–16). Even in tumors that retain wild-type *p53*, *p53* function is ultimately inhibited by the action of *MDM2* (17–19). The life cycle of the p53 protein is short; during periods of cellular stress, the p53 protein is regulated by a negative feedback mechanism. Under non-stressful conditions, p53 is regulated by the negative regulator, MDM2. Therefore, p53 and MDM2 form a feedback loop with each other and are maintained at a low level (20).

The present study demonstrated that with the intervention of curcumin, the growth of the MM RPMI 8226 cells was inhibited in a concentration- and time-dependent manner. Using qPCR to detect the mRNA expression of *p53*, *Bax* and *MDM2*, it was revealed that in the RPMI 8226 cells treated with curcumin, the expression of the *p53* and *Bax* genes was upregulated, while the expression of the *MDM2* gene was downregulated. Curcumin has the ability to upregulate *p53* and *Bax*, and downregulate *MDM2*. Since the action of *p53* is inhibited by *MDM2*, the downregulation in MDM2 may reduce the inhibition of *p53*. The results of the western blot analysis and the ELISA indicated that when 5, 10 and 15 μmol/l curcumin was administered to the MM RPMI 8226 cells for 48 h, the expression of the p53 protein was upregulated. The level of p53 protein expressed in the 15, 10 and 5 μmol/l curcumin experimental groups was higher than the amount of p53 protein expressed in the control group. Although only one MM cell line was investigated in the present study, curcumin significantly upregulated *p53* and *Bax* and downregulated the negative inhibitor for *p53*, *MDM2*. Therefore, it can be concluded that curcumin may inhibit the proliferation and induce the apoptosis of MM cells through a *p53*-mediated pathway different from the one identified in the study by Bharti *et al* (24). Further studies and the use of other MM cell lines are required in order to investigate this hypothesis and provide evidence for the clinical application of curcumin, which may provide a novel therapeutic target and an effective treatment strategy.

## Figures and Tables

**Table I:** Sequence of primers.

Target gene	Sequence of primers
*p53*	5′-CCACCATCCACTACAACTACAT-3′ 5′-AAACACGCACCTCAAAGC-3′
*Bax*	5′-TTTTGCTTCAGGGTTTCATC-3′ 5′-GACACTCGCTCAGCTTCTTG-3′
*MDM2*	5′-TACCTACTGATGGTGCTG-3′ 5′-TGATTCCTGCTGATTGAC-3′
*GAPDH*	5′-GGATTTGGTCGTATTGGG-3′ 5′-GGAAGATGGTGATGGGATT-3′

**Table II:** p53 protein content of each sample (mean ± standard deviation; n=6).

Curcumin concentration, μmol/l	p53 protein, pg/ml
0	906.035±28.324
5	1226.024±36.536
10	1302.629±40.007
15	1481.220±45.510
